# Highly efficient site-specific integration of DNA fragments into the honeybee genome using CRISPR/Cas9

**DOI:** 10.1093/g3journal/jkac098

**Published:** 2022-05-10

**Authors:** Anna Wagner, Jana Seiler, Martin Beye

**Affiliations:** Department of Biology, Institute of Evolutionary Genetics, Heinrich Heine University Düsseldorf, D-40225 Düsseldorf, Germany

**Keywords:** *Apis mellifera*, CRISPR/Cas9, homology-directed repair, methods, knock-in, gene editing

## Abstract

Functional genetic studies in honeybees have been limited to transposon mediated transformation and site directed mutagenesis tools. However, site- and sequence-specific manipulations that insert DNA fragments or replace sequences at specific target sites are lacking. Such tools would enable the tagging of proteins, the expression of reporters and site-specific amino acid changes, which are all gold standard manipulations for physiological, organismal, and genetic studies. However, such manipulations must be very efficient in honeybees since screening and crossing procedures are laborious due to their social organization. Here, we report an accurate and remarkably efficient site-specific integration of DNA-sequences into the honeybee genome using clustered regularly interspaced short palindromic repeat/clustered regularly interspaced short palindromic repeat-associated protein 9-mediated homology-directed repair. We employed early embryonic injections and selected a highly efficient sgRNA in order to insert 294 and 729 bp long DNA sequences into a specific locus at the *dsx* gene. These sequences were locus-specifically integrated in 57% and 59% of injected bees. Most importantly, 21% and 25% of the individuals lacked the wildtype sequence demonstrating that we generated homozygous mutants in which all cells are affected (no mosaicism). The highly efficient, locus-specific insertions of nucleotide sequences generating homozygous mutants demonstrate that systematic molecular studies for honeybees are in hand that allow somatic mutation approaches via workers or studies in the next generation using queens with their worker progeny. The employment of early embryonic injections and screenings of highly efficient sgRNAs may offer the prospect of highly successful sequence- and locus-specific mutations also in other organisms.

## Introduction

Honeybees are equipped with remarkable behavioral abilities, morphological, and physiological features that associate their social organization in colonies. A honeybee colony typically consists of thousands of worker bees, a single queen, and hundreds of males (drones). The worker bee caste displays a rich behavioral repertoire (Seeley 1982; Seeley and Visscher 1985; Page and Erber 2002; Johnson 2008; Robinson *et al.* 2008), sophisticated cognitive abilities (Menzel 2001, 2012), and communication abilities (Frisch *et al.* 1967; Riley *et al.* 2005) that are devoted to the maintenance of the colony. The queens display behaviors related to reproduction that include egg-laying and mating behavior. The development into either queens and workers is the outcome of female-determining and caste-determining signal (Vleurinck *et al.* 2016; Roth *et al.* 2019). The female determination signal is provided by heterozygosity at the *complementary sex determiner* (*csd*) locus (Beye *et al.*^2003^, ^2013^). The *doublesex* (*dsx*) gene is a further downstream component of the sex determination pathway regulating reproductive organ development (Roth *et al.* 2019). The differential feeding with worker diet or royal jelly during larval development determines the differentiation into either the worker and queen caste (Haydak 1970; Asencot and Lensky 1988; Kucharski *et al.* 2008; Leimar *et al.* 2012; Buttstedt *et al.* 2016; Maleszka 2018), a process that is a prominent example of developmental plasticity. A systematic dissection of the molecular processes of development and behavior in the honeybee are still limited in part due to the lack of site-specific gene manipulation tools that would enable targeted insertions of reporters and site-specific manipulations of gene functions.

So far, genes can be transgenetically expressed from endogenous and nonendogenous promoters using piggyBac-mediated transformations (Schulte *et al.* 2014; Otte *et al.* 2018). Or, endogenous genes can be site- but not sequence-specific mutated using the clustered regularly interspaced short palindromic repeats (CRISPR)/CRISPR-associated protein 9 (Cas9)-system (Kohno *et al.* 2016; Roth *et al.* 2019; Değirmenci *et al.* 2020; Chen *et al.* 2021). However, locus- and sequence-specific insertion of DNA sequences would ensure in deep analyses of genes and their molecular and organismal function. To meaningfully apply such tools in honeybees, this requires highly efficient methods, which reduce laborious screening procedures. Each reproductive female, the queen, needs to be maintained in distinct colonies together with at least few thousand worker bees in containments which is mandatory due to the genetic manipulations (Schulte *et al.* 2014; Otte *et al.* 2018).

Recent work in other species showed that providing a donor DNA together with CRISPR/Cas9 can induce homology-directed repair (HDR) resulting in the insertion of donor DNA (Gratz *et al.* 2014; Port *et al.* 2015; Hammond *et al.* 2016; Chen *et al.* 2021). This process requires that homologous sequences of a specific locus are provided to left and right of the fragment that need to be inserted. As a donor, double strand DNA (Paix *et al.* 2017) as well as single strand DNA (Quadros *et al.* 2017) can be used. Donor DNA can be a circular plasmid or a linear fragment (Gratz *et al.* 2014; Paix *et al.* 2017). The sizes of the homology arms can range between 15 bp and 1.5 kb (Gratz *et al.* 2014; Paix *et al.* 2017; Li *et al.* 2019).

In this study, we demonstrated highly efficient, locus-specific integration of DNA sequences in honeybees via CRSPR/Cas9-mediated HDR. The presented procedure offers the prospect of systematic dissection of molecular and organismal gene function and the expression of reporter genes from endogenous gene promoters. Homozygous mutants are so frequently obtained even enabling functional studies in the injected generation (Roth *et al.* 2019).

## Materials and methods

### Donor DNA, sgRNA

The 794-bp long Myc + HA DNA fragment was synthesized as a single-stranded DNA fragment (IDT Integrated DNA Technologies, Coralville, IA: Megamer Single-Stranded DNA Fragments). We composed the coding sequence as such so that 5 repeats of the c-Myc-tag (EQKLISEEDL) (Evan *et al.* 1985; Kaltwasser *et al.* 2002) and 5 repeats of the hemagglutinin (HA)-tag (YPYDVPDYA) can be expressed which we fused with a Gly–Ser–Gly (GSG) linker sequence (Supplementary Fig. 1a) (Szymczak-Workman *et al.* 2012). The *mCD8*+P2A fragment was synthesized as double-stranded molecule and was 1,229 bp long (GeneStrands, Eurofins, Ebersberg, Germany). We combined the *mCD8*, GSG linker, and 2A peptide (P2A) coding sequence (Supplementary Fig. 1b) (Szymczak-Workman *et al.* 2012). The coding sequence of the alpha chain of the mouse lymphocyte antigen CD8 was derived from the Addgene data base (Addgene plasmid # 17746; http://n2t.net/addgene:17746; RRID: Addgene_17746). We adjusted all coding sequences to the codon usage of the honeybee (*Apis mellifera*) (https://www.kazusa.or.jp/codon/cgi-bin/showcodon.cgi?species=44477).

The sequences of the homologous arms were derived from the exon 2 sequence of the *dsx* gene [NCBI; gene ID: 725126; Reference Sequence: NC_037642; Assembly: Amel_HAv3.1 (GCF_003254395.2)]. Arm lengths were ∼250 bp long, a size that gave high integration rate despite their rather small size in a previous study (Li *et al.* 2019).

The synthesized *mCD8*+P2A donor sequence was amplified using Phusion High-Fidelity DNA Polymerase (Thermo Scientific, Braunschweig, Germany) and the following oligonucleotide primers (forward primer: GTTGCAGAACGAGGAATCGGGGGAAAG; reverse primer: TGATCTTACACTTCTCGCAGGTACAAGTACG; Custom DNA Oligos, Eurofins). The amplicon was and purified with EZNA Cycle Pure kit (Omega Bio-Tek Inc., Norcross, GA) before injections.

The *dsx*-sgRNA1 was synthesized as described previously (Roth *et al.* 2019).

### Microinjection and bee handling

Fertilized honeybee eggs were injected 0–1.5 h after egg deposition (Beye *et al.* 2002; Schulte *et al.* 2014; Roth *et al.* 2019) with 53-mm injection needles (Hilgenberg, Malsfeld, Germany). Approximately 200 pg Cas9 Protein (New England Biolabs, Ipswich, MA), 18.5 pg *dsx*-sgRNA1, and donor DNA were injected into each embryo.

Rearing of hatched larvae was performed (Roth *et al.* 2019) by supplying 170 mg of the worker larval diet “Diet 7” [53% royal jelly, 4% glucose, 8% fructose, 1% yeast extract, and 34% water (Kaftanoglu *et al.* 2010; Kaftanoglu *et al.* 2011; Roth *et al.* 2019)] under restricted humidity conditions (Schmehl *et al.* 2016).

### DNA preparations, PCRs, and sequencing

Genomic DNA was isolated with the innuPREP DNA Mini Kit (Analytik Jena, Jena, Germany). PCRs were run under standard conditions (Hasselmann and Beye 2004) using Phusion High-Fidelity DNA Polymerase (Thermo Scientific) and oligonucleotide primers (forward primer: GATTCGTAATAATTCCTGTGC; reverse primer: CTTCCGCTACTCTTACTTTGAC; Custom DNA Oligos, Eurofins). For the Sanger sequencing (Mix2Seq Kit, Eurofins) amplicons were cloned into pGEM-T Easy Vector (Promega, Madison, WI).

## Results

**Fig. 1.**
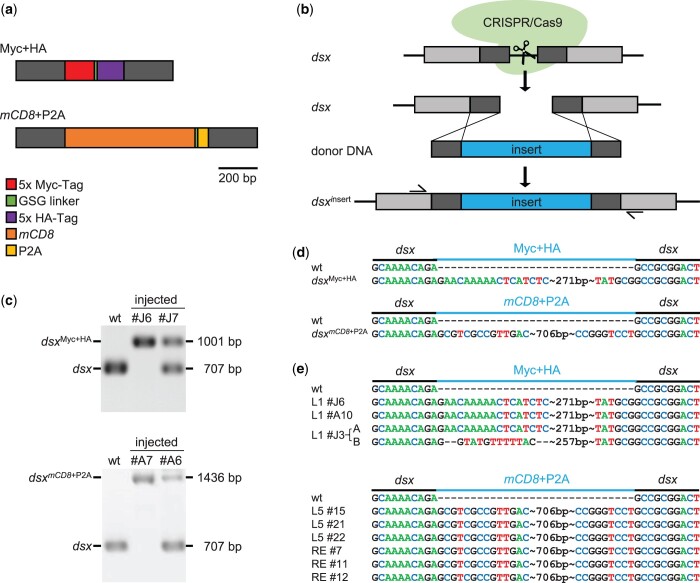
The site-specific insertion of linear DNA fragments into the *dsx* gene of the honeybee. a) Schematic presentation of the DNA fragments employed. b) Scheme of the CRISPR/Cas9-mediated HDR. The blue box indicates the new DNA fragment that needs to be integrated. The black boxes indicate the homologous arms to the left and right. Gray boxes show the remaining part of the exon. Arrows above a box indicate the position of the oligonucleotide primers for amplifications. c) Amplicons from different individuals were analyzed by size in 1% agarose gel. Black and white reversed pictures of ethidium bromide stained gels are shown. d) The expected nucleotide sequence after locus-specific insertions. wt sequences of *dsx* gene above and the expected sequence after insertion for comparison with (e). e) The detected nucleotide sequences at the target site of our homozygous mutated individuals. wt, wildtype individual (noninjected).

We used a previously published injection and CRISPR/Cas9 procedure (Schulte *et al.* 2014; Otte *et al.* 2018; Roth *et al.* 2019) of honeybees to insert 2 DNA fragments via CRISPR/Cas9-mediated HDR. The linear DNA fragment Myc+HA (Fig. 1a;Supplementary Fig. 1a) consisted of 5 repeats of a c-Myc-tag (Evan *et al.* 1985; Kaltwasser *et al.* 2002) and 5 repeats of an HA-tag (Wilson *et al.* 1984; Lee and Luo 1999), which we fused with a GSG linker (Szymczak-Workman *et al.* 2012). The other linear DNA fragment *mCD8*+P2A (Fig. 1a;Supplementary Fig. 1b) had a *mCD8* (Liaw *et al.* 1986; Lee and Luo 1999), a GSG linker and a P2A sequence (Szymczak-Workman *et al.* 2012).

To induce a homologous repair in the *dsx* gene locus (Fig. 1b) we expanded the above fragments with 250 bp to the left and to the right using the nucleotide sequences upstream and downstream of the designated cleavage site of the Cas9 protein (Fig. 1a). We selected *dsx*-sgRNA1 which has highly efficient in directing mutations using our standard procedures. This sgRNA induced in up to 100% of the injected individuals mutations (Roth *et al.* 2019). Approximately 200 pg Cas9 protein and *dsx*-sgRNA1 at a molar ratio of 1:1 together with donor DNA were injected into 0–1.5 h old embryos. We injected 20–30 pg per embryo for the Myc + HA DNA fragment and 15–20 pg for *mCD8* + P2A DNA fragment. In respect to DNA concentrations, we followed thereby results from previous donor DNA based experiments as a guideline (Schulte *et al.* 2014; Otte *et al.* 2018). Embryos were reared and bees were collected and genotyped at larvae stage. PCR amplifications at the *dsx* locus [these oligonucleotide primers were not matching sequences in our donor DNA f (Fig. 1b)] revealed that 8 out of 14 (57%) individual bees carried the *dsx*^Myc+HA^ and 40 out of 68 (59%) bees the *dsx^mCD8^*^+P2A^ allele (Fig. 1c and Table 1) suggesting a substantial integration rate. Next, we asked whether the insertions were homozygous which we examined by the presence of the inserted sequence in our bees. In 3 out 14 (21%) *dsx*^Myc+HA^ bees and in 17 out of 68 (25%) *dsx^mCD8^*^+P2A^ bees we amplified sequences with insertions (to the level of detection). This result suggests that more than 20% of the mutated bees were homozygous and that mosaicism was absent. Further, we found 5 out of 14 (36%) *dsx*^Myc+HA^ bees and 23 out of 68 (34%) *dsx^mCD8^*^+P2A^ bees with and without an insert (Fig. 1c and Table 1). The later results indicate that the DNA fragment was inserted in only 1 allele or in a subgroup of cell.

**Table 1.** The DNA fragment insertions into the *dsx* gene.

Fragment		No. of bees with			No. of mutated bees
		No insert/no insert	Insert/no insert	Insert/insert	
*dsx* ^Myc+HA^	%	43	36	21	57
	*N*	(6/14)	(5/14)	(3/14)	(8/14)
*dsx^mCD8^* ^+P2A^	%	41	34	25	59
	*N*	(28/68)	(23/68)	(17/68)	(40/68)

To validate the PCR-based genotyping results, we determined the nucleotide sequence of the amplicons from the homozygous individuals with an insert/insert genotype. Three to 11 independent clones for each of the 9 individuals were sequenced (Table 2). We found that all individuals (9 out of 9; 100%) carried the sequence of Myc + HA or *mCD8* + P2A at the designated locus (Fig. 1d and e) demonstrating targeted insertions. For 1 allele of individual J3 the sequence of the DNA fragment did not follow expectation suggesting that other mutations can rarely occur during this integration process. Thus, we conclude that 8 out 9 individuals (89%) had correctly inserted DNA sequences at the designated target site of the *dsx* gene demonstrating the power of this approach.

**Table 2.** Nucleotide sequences of the designated target site of homozygous individuals.

Fragment	Individual	No. of clones showing correct integration	
*dsx* ^Myc+HA^	L1 #J3	%	43
		*N*	(3/7)
	L1 #J6	%	100
		*N*	(7/7)
	L1 #A10	%	100
		*N*	(3/3)
*dsx^mCD8^* ^+P2A^	L5 #15	%	100
		*N*	(8/8)
	L5 #21	%	100
		*N*	(8/8)
	L5 #22	%	100
		*N*	(11/11)
	RE #7	%	100
		*N*	(5/5)
	RE #11	%	100
		*N*	(8/8)
	RE #12	%	100
		*N*	(8/8)

## Discussion

Our results demonstrate that locus-specific insertions of new sequences of more than 700 bp are now very feasible in the honeybee (Table 1). Fifty-seven percent (ss donor) and 59% (ds donor) of our injected individuals carried the insert. Eighty-nine percent of individuals had the sequence correctly inserted at the designated target site (Table 2). Furthermore, our results showed that homozygous mutants with a *dsx^insert/insert^* genotype were quite frequent in our mutated bees (Table 1 and Fig. 1e) suggesting that this technique will have broad applications for systematic molecular and organismal studies (see further below). We suggest that that at least 3 factors have substantially contributed to this efficiency that possibly can also be applied to other organisms. First, a preselected sgRNA and optimized Cas9/sgRNA concentrations that induce mutations at very high frequency, which was up to 100% of individuals in our case (Roth *et al.* 2019). Second, early embryonic injections before the first cleavage of the nucleus after 3.5 h (Schnetter 1934), which was in our case 1–3 h after egg deposition. Third, the appropriate length of the homologous sequence of our donor fragment, which was in our case 250 bp.

The efficiency of locus-specific insertions of sequences establishes the CRISPR/Cas9-mediated HDR as a powerful, new genetic tool for honeybee studies. This tool extends the existing tool box that so far consist of site directed mutagenesis (Kohno *et al.* 2016; Roth *et al.* 2019) and transposon mediated expression of transgenes (Schulte *et al.* 2014; Otte *et al.* 2018). The locus-specific insertions of Myc, HA, or *mCD8* coding sequence into the open reading frame of an endogenous gene will enable labeling of gene products in tissues which can detected by commercially available antibodies and immunostainings. Or, such tool can induce site-specific changes of nucleotides and hence amino acids that will greatly support a deep understanding of the gene’s function. This tool also offers the prospect to express molecular reporters in a subset of cells and tissues (Wang *et al.* 2019).

In *Drosophila melanogaster* the number of individuals carrying integrations is usually employed to determine integration rates, which includes individuals with mosaicism in the germline (Port *et al.* 2015). Hence, our results, which rely only on entirely mutated individuals, may indicate an even more efficient integrations in honeybees. Especially for honeybees, efficient integrations with no mosaicism are very important. Crossing experiments are laboriously in honeybees. This is because worker bees and queens need to be maintained in colonies. Genetic manipulations add further to this difficulty, since these colonies needs to be kept in a strict containment for safety reasons. These conditions limit the number of queens that can be reared and screened for the desired insertion. Now with this high insertion and homozygous rate, the methods establish a convincing approach to obtain locus- and sequence-specific manipulations in queens and hence, after instrumental inseminations, also in the worker progeny. If such next generation approach is not desirable, the high frequency and the absence of mosaicism offer the alternative route of a somatic mutation approach. We previously showed that the mutated embryos can be reared to worker bees and examined (Roth *et al.* 2019).

Studies in *D. melanogaster* reported that successful CRISPR/Cas9-mediated integration rates range between 11% (Gratz *et al.* 2014) and 26% per injected individual (Port *et al.* 2015) when using coinjected Cas9 encoding plasmids. Rates can be substantially improved up to 88% per injected individual (Gratz *et al.*^2014^, ^2015^; Port *et al.* 2015) when using transgenetically expressed Cas9 protein under the control of the *vasa* or *nos* promoters. A study in the mosquito *Anopheles gambiae* report on integration rates of 11% and 19% per injected embryo using coinjected Cas9 encoding plasmids (Hammond *et al.* 2016). Our results in honeybees now suggest that we can obtain similarly high rates by injections of Cas9 proteins without transgenetically expressing Cas9 proteins. The transgenic expression is usually not achievable for most nongenetic model organisms.

Other considerable efficiency variations within the same species can be possibly attributed to the length of the homologous arms (Li *et al.* 2019) and to the use of PCR fragments instead of donor plasmids (Paix *et al.* 2017). Lengthening the insert usually leads to reduction in the integration rate (Paix *et al.* 2017). Since our results suggest very high integration rates, it is possibly that larger inserts can also be integrated into the honeybee genome.

Hence, the technique and improvements described here, may help to develop site-specific manipulations of genomes in other organisms as well.

## Data availability

Strains and plasmids are available upon request. The authors affirm that all data necessary for confirming the conclusions of the article are present within the article, figures, and tables.

Supplemental material is available at *G3* online.

## Supplementary Material

jkac098_Supplementary_Data
