# Supplementary material for: Highly efficient site-specific integration of DNA fragments into the honeybee genome using CRISPR/Cas9
Source: G3 (Bethesda). 2022 May 10;12(6):jkac098. doi: 10.1093/g3journal/jkac098 (PMC9157169; doi:10.1093/g3journal/jkac098)
Supplement: jkac098_Supplementary_Data [file jkac098_supplementary_data.docx]

**Supplementary Data**

**Figure S1: Sequences of Myc+HA and *mCD8*+P2A.**

A) Sequence of the Myc+HA fragment. The underlined sequence represents 5x Myc tag, the italicized sequence represents GSG linker and the thick sequence represents 5x HA tag.

GTTGCAGAACGAGGAATCGGGGGAAAGAAAACTGGTGTCGAAAATCGAATCTACGCCTCGACTACGTTTCGAAACACGTGTTCTCGTTTTTTACAAGCGCGCGATAAAAGGATTAGAGAGAGAGAGAGAAAGGACAACGATAGAGGGACAAACAACCGTTCAAACATTTCATTGAGATTGTTCTTTGTAATTATGAAAAGGCTGTGAATCGAGGTTACCTATGTATCGCGAAGAGAACGAGCAAAACAGAGAACAAAAACTCATCTCGGAGGAGGATCTGGAGCAAAAGTTGATATCCGAGGAAGACCTCGAACAAAAGCTGATTTCGGAAGAAGATTTGGAGCAAAAATTGATCAGCGAGGAGGATCTCGAGCAAAAACTGATCTCCGAAGAGGACTTG*GGATCCGGA***TACCCATACGATGTTCCAGATTACGCTTACCCGTACGACGTGCCTGACTACGCATACCCTTATGATGTCCCGGACTACGCGTATCCTTACGATGTGCCTGACTACGCGTACCCTTACGACGTTCCGGATTATGCG**GCCGCGGACTTGGCTCCCCAACAACCGAGTGGTGCAAACACGTTCGAGCGTTTGGAACATTCTCAGGATAGCAAAAATGGGGACGATGGTCCCAAGAAGGTGCAAACAGACGCTTCCTCTTCGACTAATACTCCAAAGCCGCGTGCACGGAATTGTGCACGATGTCTGAATCATCGGCTGGAGATCACCTTAAAATCGCACAAGAGGTACTGCAAGTACCGTACTTGTACCTGCGAGAAGTGTAAGATCA

B) Sequence of the *mCD8*+P2A fragment. The underlined sequence represents *mCD8*, the italicized sequence represents GSG linker and the thick sequence represents Protein 2A.

GTTGCAGAACGAGGAATCGGGGGAAAGAAAACTGGTGTCGAAAATCGAATCTACGCCTCGACTACGTTTCGAAACACGTGTTCTCGTTTTTTACAAGCGCGCGATAAAAGGATTAGAGAGAGAGAGAGAAAGGACAACGATAGAGGGACAAACAACCGTTCAAACATTTCATTGAGATTGTTCTTTGTAATTATGAAAAGGCTGTGAATCGAGGTTACCTATGTATCGCGAAGAGAACGAGCAAAACAGAGCGTCGCCGTTGACGCGGTTCCTGTCGCTGAACCTGCTGCTCCTCGGTGAGTCGATTATCCTGGGTAGCGGAGAAGCTAAGCCTCAAGCACCGGAACTCCGGATCTTCCCAAAGAAAATGGACGCGGAACTCGGTCAAAAGGTGGACCTGGTATGCGAAGTGTTGGGCTCCGTTTCGCAAGGATGCTCGTGGCTCTTCCAAAaCTCCAGCTCCAAACTCCCGCAACCTACGTTCGTTGTCTACATGGCTTCGTCCCACAACAAGATCACGTGGGACGAGAAGCTGAATTCGTCGAAACTGTTCTCGGCGATGAGGGACACGAATAATAAGTACGTTCTCACGCTGAACAAGTTCAGCAAGGAAAACGAAGGCTACTACTTCTGCTCGGTCATCAGCAACTCGGTGATGTACTTCAGCTCGGTCGTGCCTGTCCTCCAAAAAGTGAACTCGACGACGACGAAGCCTGTGCTGCGGACGCCTTCGCCTGTGCACCCTACGGGAACGTCCCAACCTCAAAGGCCGGAAGATTGCCGGCCTCGGGGCTCGGTGAAGGGCACGGGATTGGACTTCGCGTGCGATATTTACATCTGGGCTCCTTTGGCGGGAATCTGCGTGGCGCTCCTGCTGTCCTTGATCATCACGCTCATCTGCTACCACTcgCGG*GGATCCGGA***GCGACGAACTTCTCGCTGTTgAAGCAAGCTGGAGACGTGGAAGAAAACCCGGGTCCT**GCCGCGGACTTGGCTCCCCAACAACCGAGTGGTGCAAACACGTTCGAGCGTTTGGAACATTCTCAGGATAGCAAAAATGGGGACGATGGTCCCAAGAAGGTGCAAACAGACGCTTCCTCTTCGACTAATACTCCAAAGCCGCGTGCACGGAATTGTGCACGATGTCTGAATCATCGGCTGGAGATCACCTTAAAATCGCACAAGAGGTACTGCAAGTACCGTACTTGTACCTGCGAGAAGTGTAAGATCA
